# Perioperative Wernicke’s encephalopathy associated with thiamine deficiency: Intraoperative EEG findings and anesthetic implications: a case report

**DOI:** 10.1097/MD.0000000000048518

**Published:** 2026-05-01

**Authors:** Yan Liu, Kejia Zhang, Tian Xie, Yue Wen, Bo Zhao, Yong Wang

**Affiliations:** a Department of Anesthesiology, The Fourth Hospital of Hebei Medical University, Shijiazhuang, Hebei, China; b Department of Neurology, The Second Hospital of Hebei Medical University, Shijiazhuang, Hebei, China; c Department of Ultrasound, The Fourth Hospital of Hebei Medical University, Shijiazhuang, Hebei, China; d Department of Medical Imaging, The Fourth Hospital of Hebei Medical University, Shijiazhuang, Hebei, China.

**Keywords:** burst suppression, electroencephalography, parenteral nutrition, perioperative management, thiamine deficiency, Wernicke’s encephalopathy

## Abstract

**Rationale:**

Wernicke encephalopathy (WE) results from thiamine deficiency and frequently affects nonalcoholic surgical patients with prolonged fasting and parenteral nutrition. Perioperative WE is often misdiagnosed due to nonspecific symptoms, and its intraoperative electroencephalographic and anesthetic features remain poorly understood.

**Patient concerns:**

A 68-year-old male underwent hepatic surgery for malignant obstructive jaundice. Postoperative biliary anastomotic leakage and aphagia led to long-term parenteral nutrition. The patient subsequently developed confusion, somnolence, nystagmus, visual loss, and peripapillary retinal hemorrhage. He also showed abnormal cerebral reactivity to anesthetics during secondary surgery.

**Diagnoses:**

Clinical diagnosis: nonalcoholic WE with thiamine-deficiency retinopathy. Infectious, septic encephalopathy and cerebral infarction were excluded.

**Interventions:**

Immediate high-dose intramuscular thiamine and magnesium supplementation were initiated. During secondary surgery, anesthesia was cautiously titrated with continuous electroencephalography and patient state index monitoring, with reduced anesthetic doses. Maintenance thiamine therapy was continued postoperatively.

**Outcomes:**

Neurological symptoms markedly improved within 3 days after thiamine treatment. Intraoperative electroencephalography revealed diffuse slowing and burst suppression under low anesthetic doses. The patient fully recovered without permanent neurological or ophthalmic sequelae and was discharged uneventfully.

**Lessons:**

Prolonged postoperative parenteral nutrition confers high WE risk in nonalcoholic surgical patients. Early empirical thiamine supplementation is essential. Thiamine deficiency increases neuronal sensitivity to anesthetics. Clinicians should enhance perioperative nutritional risk assessment and optimize anesthetic management to avoid severe cerebral complications.

## 1. Introduction

Wernicke’s encephalopathy (WE) is an acute neuropsychiatric syndrome caused by thiamine (vitamin B1) deficiency, classically presenting with confusion, ocular abnormalities, and ataxia.^[1–3]^ Although most commonly associated with chronic alcohol misuse, WE can also develop in nonalcoholic patients under conditions of increased metabolic demand, prolonged fasting, or inadequate nutritional intake.^[4–6]^ In surgical populations, especially those requiring gastrointestinal reconstruction and long-term parenteral nutrition, the risk of thiamine deficiency is often underestimated.

Although WE is widely recognized as a classical complication of thiamine deficiency, its identification and management in the perioperative setting remain challenging. Early clinical manifestations of WE are often atypical. In addition, laboratory confirmation of thiamine deficiency is not routinely performed in standard clinical practice, which may lead to delayed diagnosis. Meanwhile, the neurophysiological characteristics of WE in anesthesia-related settings have not been systematically described.^[7,8]^ Previous studies have mainly focused on neuroimaging findings or the classic clinical triad.^[9,10]^ In contrast, data on cerebral functional responses during general anesthesia in patients with WE, particularly intraoperative electroencephalographic patterns and changes in anesthetic requirements, remain scarce.

This report describes a 68-year-old male patient who developed WE after major hepatobiliary surgery, complicated by an anastomotic leak and prolonged dependence on parenteral nutrition. Although WE, as a thiamine deficiency-related complication, has been reported in multiple clinical contexts, its perioperative neurophysiological features and anesthetic responses are still insufficiently characterized. The distinctive aspect of this case lies in the presence of marked intraoperative electroencephalography (EEG) abnormalities at anesthetic doses substantially lower than standard regimens, in addition to typical neurological and ophthalmologic manifestations. These abnormalities included burst suppression (BS) and asymmetric EEG activity. Such findings suggest that thiamine deficiency may markedly increase cerebral sensitivity to anesthetic agents, reflecting the underlying vulnerability of neural metabolism. By presenting the perioperative EEG characteristics and anesthetic responses in this patient, this case provides new clinical evidence regarding the neurophysiological features of WE and its implications for anesthetic management, further expanding the current understanding of nonalcoholic postoperative WE.

## 2. Case report

A 68-year-old Han Chinese male was admitted to the hepatobiliary surgery department for a liver neoplasm complicated by malignant obstructive jaundice, with no significant past medical history. The patient underwent a series of surgical procedures, including right hemihepatectomy, cholecystectomy, and Roux-en-Y anastomosis involving the left hepatic duct and jejunum. Around 10 days post-surgery, the patient exhibited progressive abdominal distension, along with hypoproteinemia, hyponatremia, anemia, and complications from intra-abdominal infections, leading to fever. In response, a digestive tract radiography was conducted, revealing the presence of a biliary anastomotic leak. Subsequently, the patient was provided with parenteral nutrition, electrolyte fluids, and antibiotic treatment.

**Figure 1.**
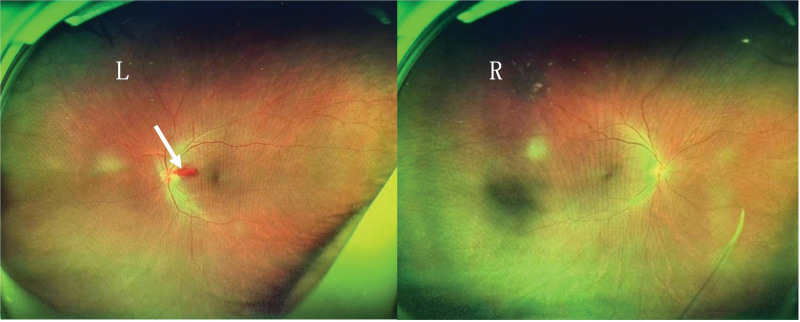
The left panel represents the ultra-wide fundus photo showing peripapillary hemorrhages in the left eye, indicated by arrows. The right panel depicts the right eye, which appears normal.

**Figure 2.**
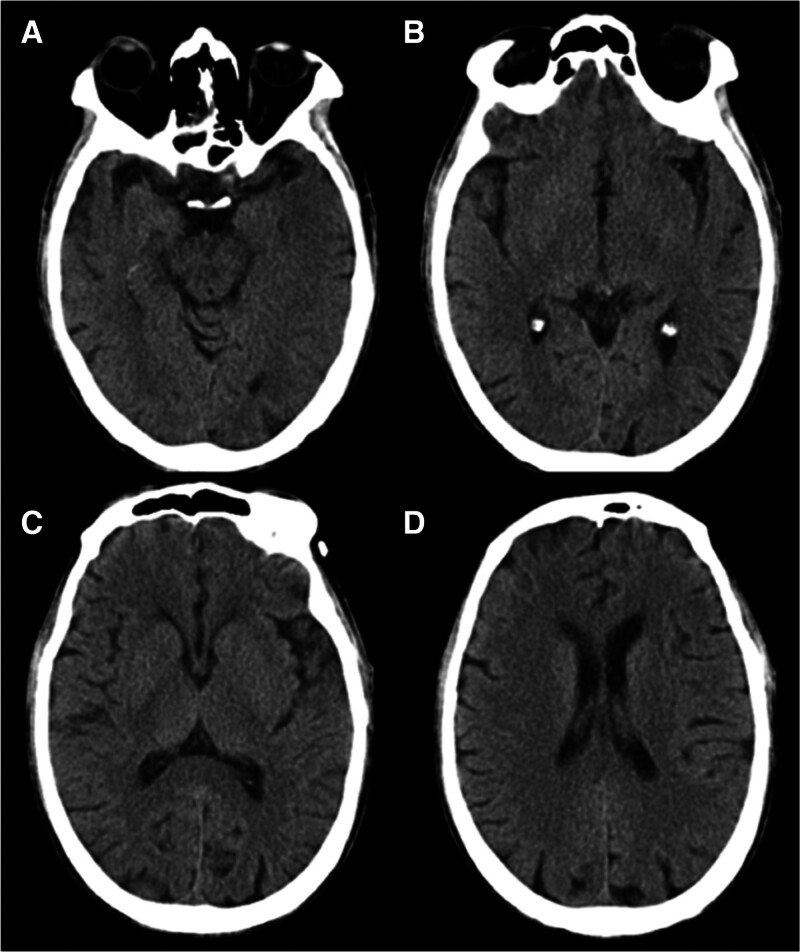
CT image of the patient. Cranial plain scan showed no abnormalities. (A) Midbrain level. (B) Third ventricle level. (C) Corpus callosum splenium level. (D) Lateral ventricle body level. CT = cranial computed tomography.

The patient’s condition deteriorated significantly 25 days after the surgery, presenting with poor mental health, somnolence, and fever. By postoperative day 35, the patient developed progressive visual deterioration accompanied by nystagmus. Best-corrected visual acuity declined to 20/500 and further deteriorated to light perception only within 2 days. Cognitive impairment became evident as the patient struggled with verbal communication and was unable to provide accurate responses to questions. Notably, there were no instances of nausea, vomiting, or adverse physical manifestations. A thorough neurological examination was conducted by a neurologist. Neurological examination revealed delayed responses, dysarthria, and spatial disorientation. During the sensory and ataxia evaluation, the patient demonstrated uncooperative behavior. Bilateral Babinski signs were positive, whereas Kernig and Brudzinski signs were negative. Both funduscopy and brain cranial computed tomography scans were performed. The funduscopy indicated peripapillary intraretinal hemorrhages specifically on the left optic disc (Fig. 1), with no other notable abnormalities (Fig. 2). Vascular and degenerative changes were effectively ruled out as potential causes.

Taking into account the progression of the patient’s medical history and the clinical findings, the neurologist identified a potential vitamin B1 deficiency as the underlying cause of the acute presentation of WE. Although cranial magnetic resonance imaging and vascular imaging were considered during the diagnostic process, these examinations were not completed promptly because the patient was critically ill and required urgent reoperation. As an immediate response, intramuscular administration of 200 mg of vitamin B1 was initiated 4 times a day (Q.I.D). The impact of this intervention was striking, as the patient exhibited a significant improvement on the third day of thiamine therapy. Notably, the patient’s visual acuity was restored to 20/500; however, their mental state had yet to demonstrate improvement. Table 1 succinctly summarizes the noteworthy shifts observed in the laboratory data. Furthermore, there was a discernible decrease in magnesium concentration around the time of WE onset. To address this deficiency, intravenous administration of 1.25 mg of magnesium sulfate Q.I.D. was administered to achieve the desired therapeutic effect.

**Table 1:** Blood chemistry of our patients during the perioperative period.

Parameter	Preoperative	POD 1	POD 7	POD 25	POD 35	POD 40 (second surgery)	POD 47	Reference range
White blood cells (×10^9^/L)	4.42	**17.60↑**	**16.68↑**	5.42	**3.68↓**	**2.99↓**	5.01	4.0–11.0
Red blood cells (×10^12^/L)	**3.61↓**	**3.25↓**	**3.48↓**	**2.82↓**	**2.7↓**	**2.34↓**	**2.98↓**	4.7–6.1 (M); 4.2–5.4 (F)
Hemoglobin (g/L)	**122↓**	**106.3↓**	**112.4↓**	**90↓**	**86↓**	**75↓**	**92.4↓**	135–175 (M); 120–155 (F)
Total protein (g/L)	60.9	**48.2↓**	**51.7↓**	**52.7↓**	**50.7↓**	**54.2↓**	**57.4↓**	60–80
Albumin (g/L)	37.4	**32.7↓**	**32.8↓**	**29.6↓**	**31.8↓**	**32.7↓**	**33.2↓**	35–50
AST (U/L)	**92.1↑**	**1173.4↑**	**75.4↑**	**52.1↑**	**42↑**	16.7	17.4	10–40
ALT (U/L)	**70.2↑**	**1256.3↑**	**150.7↑**	43.7	**5.1↓**	19.7	29.8	7–56
Creatinine (µmol/L)	66.5	**154.6↑**	73.1	**49.8↓**	**42.0↓**	55.6	**48.9↓**	62–106 (M); 44–80 (F)
BUN (mmol/L)	4.9	**11.9↑**	**13.5↑**	**7.3↑**	5.1	**7.6↑**	**7.8↑**	2.5–7.1
Cholesterol (mg/dL)	97.46	67.6	**35.1↓**	**28.95↓**	**33.5↓**	**15.42↓**	**28.17↓**	<200
Sodium (mmol/L)	137.8	136.3	**131.8↓**	**128.1↓**	**132.9↓**	**146.4↑**	142.3	135–145
Potassium (mmol/L)	4.35	4.74	4.32	4.89	**3.11↓**	3.97	**3.47↓**	3.5–5.0
Chloride (mmol/L)	101.2	102.6	98.1	**95.5↓**	98.2	**116.8↑**	98.2	96–106
Magnesium (mmol/L)	0.86	**0.62↓**	**0.50↓**	**0.24↓**	**0.18↓**	**0.55↓**	0.74	0.7–1.0
Calcium (mmol/L)	2.27	**2.05↓**	**2.01↓**	**2.08↓**	**1.81↓**	**2.12↓**	**4.43↓**	2.2–2.6
Glucose (mmol/L)	4.43	3.96	6.17	4.45	**8.45↑**	**7.33↑**	**7.07↑**	2.2–2.6

Bold values indicate the abnormal results.

ALT = alanine aminotransferase, AST = aspartate aminotransferase, BUN = blood urea nitrogen, F = female, M = male, POD = postoperative day.

**Figure 3.**
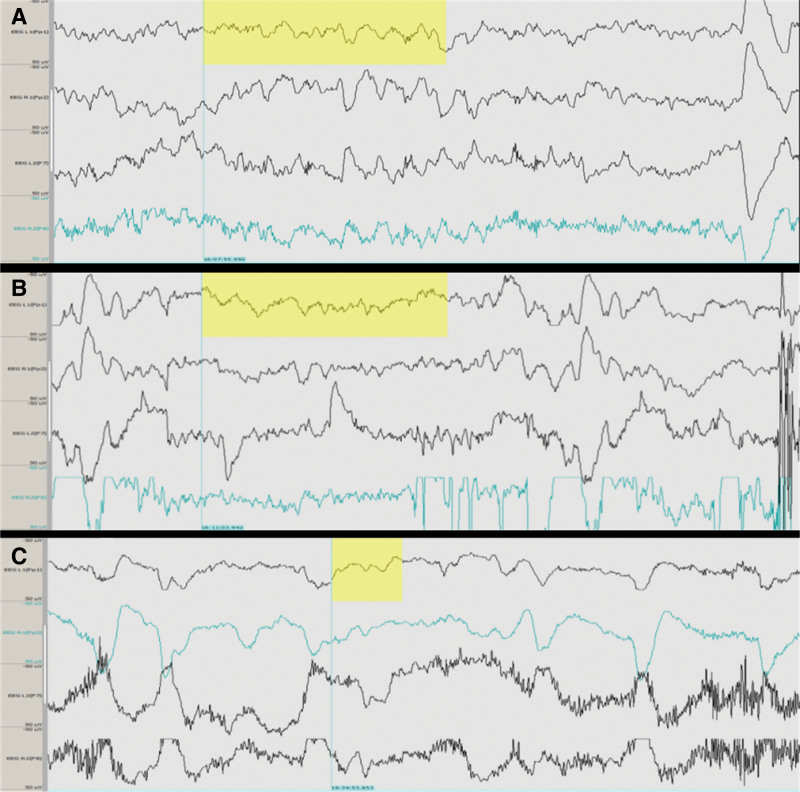
Electroencephalogram of patients at different times. Nonspecific background slow waves appeared in the waking state (yellow area). (A) Pre-anesthesia nonspecific slow waves. (B) Nonspecific slow waves during the anesthesia recovery period. (C) Nonspecific slow waves in the waking state. Each panel illustrates the different states of brain activity, with the nonspecific background slow waves highlighted in the yellow areas.

A re-operation became necessary due to the occurrence of a biliary anastomotic leak. In the operating room, the patient displayed inaccurate responses to questions, along with signs of drowsiness and ataxia. The patient struggled to use the tip of their nose effectively. Subsequently, a Sedline Brain Function electrode was utilized, revealing a patient state index (PSI) significantly below the norm at approximately 50. For induction of general anesthesia, the patient was administered sufentanil 25 µg, etomidate 20 mg, and cisatracurium 12 mg. EEG monitoring revealed BS, with a burst suppression ratio of 12% and a PSI of 17, prompting adjustment of the target anesthetic depth to a PSI range of 25 to 50. To maintain a suitable depth of anesthesia, post-burst suppression ratio normalization, a mere 0.4 minimum alveolar concentration sevoflurane, and approximately 0.05 to 0.1 µg kg^−1^ min^−1^ remifentanil was administered. Notably, significant EEG abnormalities were observed at sedative and analgesic doses well below those used in standard anesthetic protocols. These changes included a marked reduction in the PSI and the appearance of BS. Such doses are typically insufficient to induce BS in patients without encephalopathy. Additionally, norepinephrine at a rate of 0.02 to 0.05 µg kg^−1^ min^−1^ was employed to sustain adequate hemodynamics. Notably, instances of asymmetrical BS in bilateral cerebral hemispheres were intermittently recorded. During the patient’s waking state, the EEG exhibited nonspecific background slow waves (Fig. 3). To facilitate a prompt recovery, sevoflurane administration ceased 30 minutes before surgery conclusion, leading to the patient regaining consciousness within 10 minutes. Despite the second operation, the patient persisted with confusion and somnolence. Continued treatment encompassed intramuscular administration of 200 mg of vitamin B1 and intravenous infusion of 1.25 mg of magnesium sulfate Q.I.D. Over the ensuing 10 days, the patient’s neurological symptoms began to ameliorate. Mental clarity was restored, evidenced by coherent verbal communication, while the somnolence dissipated without any recent memory impairment. Addressing mild hypomagnesemia and hypoproteinemia, interventions led to a serum magnesium concentration of 0.74 mmol/L. Upon discharge from the hospital, the patient exhibited no enduring sequelae stemming from the vitamin deficiency. During follow-up visits, no instances of intraoperative awareness or lasting impairment were clinically observed. No adverse or unanticipated events occurred. Perioperative monitoring did not reveal significant hypotension, hypoxemia, hypercapnia, hypothermia, or hypoglycemia, thereby reducing the likelihood that these physiological factors contributed to the observed EEG abnormalities. To facilitate a comprehensive understanding of the perioperative clinical course and the temporal relationship among key events, major clinical processes and laboratory parameter changes are summarized chronologically (Table 2).

**Table 2:** Chronological summary of the patient’s perioperative clinical course.

Time point	Main event	Management	Examination findings	Laboratory tests	
				Magnesium (mmol/L)	Albumin (g/L)
Hospital admission	Admission for hepatic mass	–	–	0.86	37.4
Day of surgery	Right hemihepatectomy, cholecystectomy, left hepatic duct jejunum Roux-en-Y anastomosis	Standard perioperative management	–	0.70	32.7
POD 10	Abdominal distension after meals; biliary-enteric anastomotic leakage	Abdominal catheter drainage, fasting, parenteral nutrition, fluid replacement, and anti-infective therapy	–	0.50	32.8
POD 20	Poor mental status, drowsiness, and fever	Correction of electrolyte imbalance and hypoalbuminemia; continued anti-infective therapy	–	0.39	31.1
POD 35	Decreased vision accompanied by nystagmus	Ophthalmology consultation; fundoscopic examination	Peripapillary retinal hemorrhage; best-corrected visual acuity decreased to 20/500	0.18	31.8
POD 38	Apathy, spatial disorientation, dysarthria; vision further decreased to light perception only	Neurology consultation, neurological examination, suspected Wernicke encephalopathy, Vitamin B1 200 mg intramuscular injection 4 times daily	Bilateral Babinski signs positive	0.21	31.0
POD 42	Visual acuity recovered to 20/500; no significant improvement in mental status	Vitamin B1 200 mg intramuscular injection 4 times daily	–	0.39	32.2
POD 40	Jejunojejunostomy	Maintenance of appropriate anesthetic depth using low-dose anesthetic agents	Intraoperative EEG showed asymmetric burst suppression in both cerebral hemispheres; background slow waves observed before anesthesia and during recovery	0.61	32.1
POD 47	Mental status recovered; speech clear and coherent; drowsiness resolved	Vitamin B1 discontinued	–	0.74	31.1
POD 90	Discharged for rehabilitation	–	–	0.66	35.3

EEG = electroencephalography, POD = postoperative day.

## 3. Family perspective

Before the initial surgery, my father had no preexisting medical conditions. After the procedure, however, he developed a serious complication, namely bile–jejunal anastomotic leakage. Due to prolonged fasting and subsequent malnutrition, he gradually experienced marked visual impairment and altered mental status approximately 1 month later. At that time, our family feared that he had suffered a cerebral infarction. Fortunately, the attending physicians made the correct diagnosis of WE promptly. After thiamine (vitamin B1) supplementation was initiated, his condition improved rapidly. Although he later underwent 2 additional surgical procedures to manage the anastomotic leakage, he ultimately achieved full recovery and was discharged in good condition. To date, he has not developed any permanent ophthalmologic or neurological sequelae.

## 4. Discussion

WE is a well-recognized but often underdiagnosed complication of thiamine deficiency.^[8,11,12]^ While alcohol misuse remains the most common etiology, increasing evidence indicates that nonalcoholic patients, particularly those undergoing gastrointestinal surgery and prolonged parenteral nutrition, are also at risk.^[13–18]^ The perioperative setting poses additional diagnostic challenges because symptoms such as confusion, drowsiness, or visual disturbance may easily be attributed to postoperative complications, infection, or metabolic derangements rather than nutritional deficiency.^[1,2,7,19–24]^ Consequently, delayed diagnosis is common and may result in irreversible neurological injury. In this context, beyond discussing the diagnosis and etiology of WE, this case also provides informative perioperative observations. These findings are particularly evident in the abnormal intraoperative EEG patterns and the markedly increased sensitivity to anesthetic agents. Such observations offer a new perspective for understanding neurophysiological suppression associated with thiamine deficiency and its implications for perioperative clinical management.

The diagnosis of WE primarily relies on clinical recognition, as no single laboratory test or imaging modality serves as a diagnostic gold standard. The operational criteria proposed by Caine et al are widely applied in clinical practice. In patients with nutritional risk factors, a diagnosis of WE is supported when at least 2 of the following 4 features are present: inadequate dietary intake or evidence of thiamine deficiency, oculomotor abnormalities, cerebellar dysfunction, and altered mental status or memory impairment.^[25]^ In this case, the patient experienced prolonged fasting and dependence on parenteral nutrition following gastrointestinal surgery, indicating a clear nutritional risk. He also developed nystagmus, visual loss, and impaired consciousness, fulfilling the Caine diagnostic criteria. Regarding differential diagnoses, hepatic encephalopathy was considered unlikely because liver function indices gradually improved, and neurological symptoms did not parallel hepatic fluctuations. Septic encephalopathy was also less likely, as postoperative white blood cell counts normalized and neurological deterioration persisted despite infection control. Metabolic disturbances, including electrolyte abnormalities, were corrected without corresponding neurological improvement. In contrast, rapid clinical recovery following high-dose thiamine therapy strongly supported the diagnosis of WE.

Electroencephalographic abnormalities have been reported in WE but remain insufficiently investigated in a systematic manner. Previous studies indicate that EEG changes often correlate with clinical severity. Frantzen et al reported that, with disease progression, EEG findings may evolve from diffuse background slowing to low-voltage theta (4–8 Hz) and delta (0.5–4 Hz) activity, sometimes accompanied by reduced responsiveness to external stimuli.^[26]^ Martínez et al further observed predominant frontotemporal slow-wave activity in patients with Wernicke syndrome, suggesting widespread cortical suppression.^[27]^ Under physiological conditions, delta and theta waves are typically observed during deep sleep, early infancy, generalized cerebral depression, or anesthesia. In the present patient, diffuse background slowing was already evident in the awake state, indicating significant metabolic cerebral dysfunction. During low-dose anesthesia, asymmetric BS was observed between the bilateral cerebral hemispheres, suggesting that cortical inhibition was not fully synchronous. A possible explanation is regional variability in metabolic vulnerability under thiamine-deficient conditions, resulting in uneven reductions in neuronal excitability. Although technical factors or occult structural lesions cannot be entirely excluded, the absence of focal neurological deficits before surgery and the lack of structural abnormalities on cranial computed tomography support a metabolic origin. Moreover, BS is generally regarded as a marker of deep anesthesia or severe cerebral suppression. Its occurrence at extremely low anesthetic doses further suggests that thiamine deficiency markedly reduces cerebral tolerance and anesthetic requirements.

Ocular manifestations are a classical feature of WE. Previous reports have mainly focused on nystagmus, ophthalmoplegia, and visual impairment, whereas retinal hemorrhage has been described far less frequently.^[28–30]^ Existing literature contains only a limited number of cases reporting thiamine deficiency-related retinal or optic nerve changes, and peripapillary intraretinal hemorrhage is not a common finding. Therefore, the fundoscopic changes observed in this patient may represent a rare or atypical form of ocular involvement. In terms of differential diagnosis, the patient did not exhibit perioperative thrombocytopenia or coagulation abnormalities. There was no history of anticoagulant or antiplatelet therapy. In addition, hypertensive crisis and diabetic retinopathy were not observed. These factors reduce the likelihood that hematologic disorders or common vascular causes were responsible for the retinal hemorrhage.

Traditionally, WE is considered to predominantly affect oculomotor function. However, studies examining thiamine deficiency in the context of diabetic retinopathy have shown that thiamine depletion can induce retinal injury through 2 major mechanisms: energy failure and oxidative stress–mediated apoptosis. Retinal pigment epithelial cells and retinal ganglion cells are highly dependent on oxidative glucose metabolism for survival and function. Thiamine pyrophosphate (TPP), the active form of thiamine, is an essential cofactor for pyruvate dehydrogenase, α-ketoglutarate dehydrogenase, and transketolase. When TPP is deficient, glucose metabolism is impaired, leading to ATP depletion, disrupted renewal of photoreceptor outer segments, and reduced retinal pigment epithelial pump function. These changes ultimately result in intracellular edema and ionic imbalance.^[31]^ In addition, metabolic blockade promotes the accumulation of lactate and reactive oxygen species. Simultaneously, inhibition of the pentose phosphate pathway reduces nicotinamide adenine dinucleotide phosphate production, weakens antioxidant capacity, and triggers mitochondrial-mediated apoptosis. Animal studies have demonstrated vacuolar changes in the retinal ganglion cell layer, axonal swelling, and disruption of photoreceptor outer segments under thiamine-deficient conditions. Consistent with these mechanisms, the present patient developed retinal abnormalities in parallel with WE. These findings underscore the importance of routine fundus examination in this patient population.

The present case highlights several clinically important issues. First, it illustrates how thiamine deficiency and subsequent WE can develop rapidly after hepatobiliary surgery complicated by anastomotic leakage and extended parenteral nutrition. Second, it provides unique intraoperative EEG findings, with BS occurring under unusually low anesthetic doses, suggesting increased cerebral vulnerability in thiamine-deficient states. Finally, the case underscores the importance of early recognition and prompt thiamine supplementation, which resulted in rapid neurological improvement and complete recovery in our patient.

Previous reports have emphasized that WE after gastrointestinal surgery often presents atypically and can be mistaken for other postoperative complications. In a review of nonalcoholic WE, ocular abnormalities such as nystagmus or visual deterioration were frequently the initial manifestations, which is consistent with our patient’s presentation.^[32,33]^ Retinal hemorrhages, as observed in this case, have been rarely described but may represent an early indicator of severe thiamine deficiency.

From an anesthetic perspective, this case is particularly noteworthy. Intraoperative monitoring demonstrated background slow waves and BS at unusually low anesthetic doses. BS is generally considered a marker of deep anesthesia, cerebral hypometabolism, or severe encephalopathy. The occurrence of BS with minimal anesthetic exposure suggests that thiamine deficiency may significantly alter neuronal excitability and reduce anesthetic requirements. This observation has important implications for anesthetic management in patients with metabolic encephalopathies: patients with WE or other metabolic encephalopathies may be highly sensitive to anesthetic agents, and reliance on standard dosing protocols could increase the risk of excessive cerebral depression.

In addition, this case reinforces the role of electrolyte balance, particularly magnesium, in the treatment of WE. Hypomagnesemia was observed in our patient at the time of neurological deterioration, and magnesium supplementation was provided alongside thiamine therapy. Previous studies have shown that magnesium acts as a cofactor for thiamine-dependent enzymes and that deficiency can impair the clinical response to thiamine replacement.^[34]^ Therefore, both thiamine and magnesium levels should be considered in perioperative nutritional management.

From a biochemical perspective, magnesium plays a critical role in thiamine-dependent metabolic pathways. Magnesium ions are required not only for the formation of TPP but also for the structural integrity and catalytic activity of multiple TPP-dependent enzymes, including pyruvate dehydrogenase, α-ketoglutarate dehydrogenase, and transketolase. In states of severe hypomagnesemia, the conversion of thiamine to its active form and subsequent enzymatic reactions may remain significantly impaired, even when thiamine supplementation is adequate. In this case, the patient developed profound hypomagnesemia, which likely exacerbated thiamine deficiency-related energy metabolism failure. Magnesium depletion can further reduce mitochondrial ATP production and increase the instability of neuronal membrane potentials. These effects may heighten cortical neuronal sensitivity to metabolic suppression and anesthetic agents. This “dual metabolic insult” may explain why pronounced EEG abnormalities, including background slowing and BS, occurred under low-dose anesthesia. Recent studies have emphasized the amplifying role of magnesium deficiency in the perioperative development and progression of WE. These findings indicate that hypomagnesemia significantly weakens the recovery of thiamine-related metabolic pathways and is associated with more severe neurological manifestations.^[35]^ Therefore, perioperative nutritional management should address not only thiamine supplementation but also prompt identification and correction of magnesium deficiency.

The limitations of this report should also be acknowledged. Laboratory measurement of thiamine and its phosphate esters was not performed, and the diagnosis relied on clinical presentation and response to therapy. While this approach is consistent with established diagnostic criteria, biochemical confirmation could have provided additional support. Moreover, as a single case report, the generalizability of the intraoperative EEG findings is limited, and further studies are required to clarify the mechanisms linking thiamine deficiency with altered anesthetic sensitivity.

In summary, this case underscores the importance of early recognition and aggressive treatment of thiamine deficiency in surgical patients with prolonged parenteral nutrition. Of particular note, this patient developed marked intraoperative EEG abnormalities, including BS, at anesthetic doses well below standard regimens. Such findings have rarely been reported in previous cases of WE. This observation suggests that thiamine deficiency may substantially lower the cerebral response threshold to anesthetic agents, reflecting underlying neuro-metabolic vulnerability. These results extend current knowledge of perioperative neurophysiological features in WE and carry direct clinical implications. When nonalcoholic gastrointestinal surgical patients develop unexplained neurological symptoms in the perioperative period, clinicians should maintain a high index of suspicion for WE. Anesthesiologists should also account for heightened anesthetic sensitivity in high-risk patients when planning anesthetic strategies and adjusting anesthetic depth, in order to reduce the risk of excessive cerebral suppression.

## 5. Conclusions

This case illustrates that WE can develop in nonalcoholic surgical patients receiving prolonged parenteral nutrition, and that its manifestations may mimic other postoperative complications. Early recognition and prompt thiamine supplementation are essential to prevent irreversible neurological injury. In addition, our report highlights novel intraoperative EEG findings under low-dose anesthesia, suggesting increased cerebral vulnerability in thiamine-deficient states. Surgeons and anesthesiologists should remain vigilant for nutritional deficiencies in the perioperative setting and tailor anesthetic management accordingly.

## Author contributions

**Supervision:** Yong Wang.

**Funding acquisition:** Yan Liu.

**Methodology:** Yan Liu.

**Writing – original draft:** Yan Liu.

**Data curation:** Kejia Zhang, Tian Xie, Yue Wen, Bo Zhao.

**Writing – review & editing:** Kejia Zhang, Tian Xie, Yue Wen, Bo Zhao.
